# Design and Implementation of a MAC Protocol for Timely and Reliable Delivery of Command and Data in Dynamic Wireless Sensor Networks

**DOI:** 10.3390/s131013228

**Published:** 2013-09-30

**Authors:** Hoon Oh, Phan Van Vinh

**Affiliations:** Ubicom Lab, School of Computer Engineering and Information Technology, University of Ulsan, P.O. Box 18, Ulsan 680-749, Korea; E-Mails: hoonoh@ulsan.ac.kr (H.O.); pvvinhbk@gmail.com (P.V.); Tel.: +82-52-259-1257; Fax: +82-52-259-1687

**Keywords:** reliable link, TDMA, slot scheduling, monitoring and control, bidirectional communication

## Abstract

This paper proposes and implements a new TDMA-based MAC protocol for providing timely and reliable delivery of data and command for monitoring and control networks. In this kind of network, sensor nodes are required to sense data from the monitoring environment periodically and then send the data to a sink. The sink determines whether the environment is safe or not by analyzing the acquired data. Sometimes, a command or control message is sent from the sink to a particular node or a group of nodes to execute the services or request further interested data. The proposed MAC protocol enables bidirectional communication, controls active and sleep modes of a sensor node to conserve energy, and addresses the problem of load unbalancing between the nodes near a sink and the other nodes. It can improve reliability of communication significantly while extending network lifetime. These claims are supported by the experimental results.

## 1. Introduction

Context-aware safety monitoring and control applications that collect data from the target environment using Wireless Sensor Networks (WSNs), analyze the collected data, and judge the situation of the environment are in a growing need in industrial fields. One of the key technologies to enable such applications is a WSN that can ensure reliable data transmission. However, the industrial environment is often non-friendly to wireless communication and the topology of the WSN deployed in that environment tends to change dynamically. Thus, it is quite challenging to apply the Time Division Multiple Access (TDMA) technology. Furthermore, if sensor devices are installed on the high walls of working places, the replacement of a battery is cumbersome. Thus, a protocol needs to address the balancing of power consumption as well as the power management of individual sensor devices to lengthen network lifetime.

Most of earlier contributions on the design of the MAC protocol focused on low-duty cycle applications to reduce energy consumption. S-MAC [1] with an active-sleep cycle forces nodes to sleep periodically in the idle listening period to conserve energy. S-MAC also uses collision and overhearing avoidance techniques which allow nodes that overhear other transmissions to sleep immediately to reduce energy consumption. However, a static active-sleep cycle of S-MAC can result in high latency and low throughput in variable traffic loads. T-MAC [2] is proposed to enhance the drawbacks of S-MAC by using an adaptive active-sleep cycle, where the listen period ends if no activation event happens for a threshold time. B-MAC [3] employs the Clear Channel Assessment (CCA) technique to improve channel utilization and the Low Power Listening (LPL) scheme with the adaptive preamble sampling to reduce duty cycle and minimize idle listening period. However, because of using the long preamble mechanism, the packet latency is gradually accumulated when packet travels through multi-hop paths and energy is wasted at both the sender and receiver after the receiver has woken up. CMAC [4] is proposed to mitigate the latency in B-MAC by using an aggressive Request to Send (RTS) instead of the long preamble for channel assessment. However, network overhead increases due to the use of an anycast mechanism. DMAC [5] tries to solve the data forwarding interruption problem in S-MAC by using a staggered wakeup schedule to assign time slots for all nodes on the multi-hop path to wake up sequentially. It also proposed a data prediction mechanism and the use of more-to-send packets in order to alleviate problems pertaining to channel contention and collision. Because no collision avoidance technique is used, collisions may occur when nodes that have the same schedule (same level in tree) try to send data to the same node. In summary, most of early MAC protocols designed for low duty applications improve energy efficiency, but increase latency and end-to-end delay as well because they use an active-sleep cycle.

Recently, several TDMA-based MAC protocols were proposed since contending for the medium is one of the main sources of delays. TRAMA [6] employs a distributed election scheme that is based on traffic information at each node to determine which node can transmit in a particular time slot. LMAC [7] divides the time interval into 32 slots for assignment to nodes. CR-SLF [8] employs a centralized scheduling algorithm with spatial channel reuse. Each message is associated with the deadline and the start time of the consuming task at the destination, and the messages are partitioned into disjoint sets so that they can be transmitted in parallel without interference. PR-MAC [9] employs the Bidirectional Pipelining Schedule (BPS) algorithm which enables a node to wake up twice during a work cycle to support bidirectional data transmission and uses a multi-channel mechanism to improve network throughput. In either direction, the nodes along a path wake up sequentially in the way of pipelining with some offset which is long enough for transmitting or receiving a packet. However, PR-MAC needs time synchronization. RRMAC [10] tries to reduce end-to-end delay by assigning time slots in a sequence so that the packets flow continuously from leaf-level nodes to the top-level nodes in a tree topology. RT-MAC [11] focuses on maximizing spatial channel reuse by guaranteeing simultaneous transmission of packets with no collision for the nodes separated by four hops. RT-MAC uses predictable feedback to avoid collisions such that the first data packet is transmitted adventurously, but the transmission of subsequent data packets depends on the successful progression of their previous data packets. RT-MAC also requires global time synchronization.

Some approaches combine the advantages of TDMA-based and CSMA-based protocols. Z-MAC [12] uses DRAND [13] to allocate time slots to every node such that no two nodes within two-hop neighbors are assigned the same time slot in order to prevent interference. However, due to its overhead, they recommend the execution of DRAND only at the network initialization time. Z-MAC also employs the CSMA scheme to steal the slots in case that a node does not use its own slots occasionally due to the variation of data traffic. Funneling-MAC [14] responds to the funneling effect that takes place because nodes close to a sink have to process much more data packets delivered from their descendants. Meanwhile, some tree-based protocols were proposed for wireless *ad hoc* networks [15–17] and wireless sensor networks [18–21]. TreeMAC [18], SDA [19], DAS [20], and WIRES [21] focus on generating data transmission schedule to increase channel spatial reuse. In the latter three approaches, every node is assigned a single big slot enough to transmit the largest aggregated data. However, it is not easy to determine a slot size since this varies according to the largest number of descendants that a node has. Furthermore, it may not be efficient or practical to assign one big slot to each node per one data collection round from the viewpoint of bandwidth utilization. TreeMAC defines a superframe (SF) which every node in a tree can send data to a sink. It divides the SF into a number of frames, each consisting of three slots. Then, a node assigns a sequence of frames to each of its children based on the relative bandwidth demand, and each node determines its sending and receiving slot number within the assigned frames such that an identical slot number is not selected among the nodes located within a vertical two-hop distance. In this way, it eliminates the vertical two-hop interference, thus enabling slot reuse. GinMAC [22], a TDMA based MAC protocol, is proposed to provide reliable data delivery for time critical applications in tree topology networks. GinMAC time frame consists of three types of slots: basic slots, additional slots and unused slots. Within a time frame, each sensor can forward one message to the sink and the sink can transmit one message to each actuator. GinMAC uses exclusive slots to mitigate the interference problem. However, GinMAC is designed for fixed small networks. Furthermore, it may increase packet delivery latency since it uses additional slots and unused slots for reliability of transmission.

Most of the TDMA-based MAC protocols discussed so far have focused on devising a slot scheduling algorithm and increasing slot reuse or channel efficiency. However, slot reuse often incurs irregular interference. According to the theory and measurement about radio propagation [1], the received signal power *P_r_* decreases with the distance *d* since *P_r_* α *P_t_d^−β^*, where *P_t_* is the transmission power and *β* is an environment-dependent constant normally between 2 and 5. Since the transmission signal disappears gradually as the distance increases, an interference range of radio signal is always farther than a transmission range. Furthermore, interference becomes worse with node mobility, making it difficult to achieve a reliable communication with a slot scheduling scheme. Fortunately, according to our previous study [23], channel efficiency gain by slot reuse is trivial in a small sized network that can be used for the safety monitoring and control application. In addition, the existing protocols suffer from the imbalance of power consumption since the nodes closer to a sink are assigned more slots or remain active longer. The Filtering and Aggregation (FA) technique can be used to alleviate this problem. However, since TreeMAC alternates a receiving slot and a sending slot within a frame, the FA technique cannot be employed. Lastly, a MAC protocol for this type of application should be able to provide the capability of a bidirectional communication. Therefore, in this paper, we propose a new MAC protocol, named I-MAC, which is based on TDMA for safety monitoring and control applications of a small size network. We discuss experimental results on the testbed using TelosB and evaluate our protocol comparatively with other well-known protocols. In summary, the main contributions of this paper are as follows:

- We identify the problems of the existing MAC protocols, and in particular we analyze the effect of slot reuse in small control networks.
- For reliable communication, we propose a scheme to build a tree topology that has bi-directionally reliable links. Moreover, we use MAC control messages for ensuring reliable data transmission within every slot.
- We propose a protocol that enables bidirectional communication, ensures timely and reliable delivery of data and command in dynamic WSNs.
- We present a spare time utilization scheme in which a node can use the slots that have been assigned to its neighboring nodes under the grant of the owner.
- We show by experiments on TelosB sensor testbed that the proposed protocol enables the high reliability of data transmission and significantly reduces energy consumption at the nodes close to a sink, thus enables the balancing of power consumption and extends network lifetime.

The remaining part of this paper is organized as follows: Section 2 gives the background of this research. In Section 3, we present the formal description of the proposed protocol with the solutions for some derived problems. Section 4 explains the test-bed and evaluates the protocol with experimental results. Finally, we provide concluding remarks in Section 5.

## 2. Background

### 2.1. Network Model

A series of Liquefied Petroleum Gas (LPG) tanks are built inside an LPG tank that has to be kept at the extremely low temperature, less than −47 °C. In this building process, people perform work to shield the outer wall of a tank with some thermal insulation materials such as styrofoam, urethane, or glass wool, *etc.* and also perform welding jobs after entering the deep insides of the ship. During the welding job, people can be faced with the risk of fire of the thermal insulation material due to the sparks or can be suffocated due to the emission of poisonous gas or smoke. Since the dimension of the container in which one LPG tank is installed is approximately 20 m × 30 m, a small network with about thirty nodes and the transmission range of 5 to 10 m can cover one container for safety monitoring. Each sensor communication device can be integrated with various sensor modules such as a thermal sensor module, a gas sensor module, an oxygen sensor module, a smoke sensor module, and a flame sensor module that have different power consumption level. They obtain data from environment periodically and send the data to a server via a WSN. The server collects data and analyzes the data to judge whether the target environment is safe or not. If it detects a dangerous situation, it warns the workers of the situation. This kind of system is referred to as a Safety MOnitoring and Control System (SMOCS). Since sensor communication devices can be either installed onto some spots of the wall, or carried by the workers, network topology may change. In addition, the environment is harsh or non-friendly to the activity of communication due to the communication obstacles such as steel materials and the structure changing over construction time. Furthermore, the recharge or replacement of the battery is not easy if it is installed on a high wall. Thus, it is desirable that the network lasts for four months, which is a typical ship construction time.

**Figure 1.**
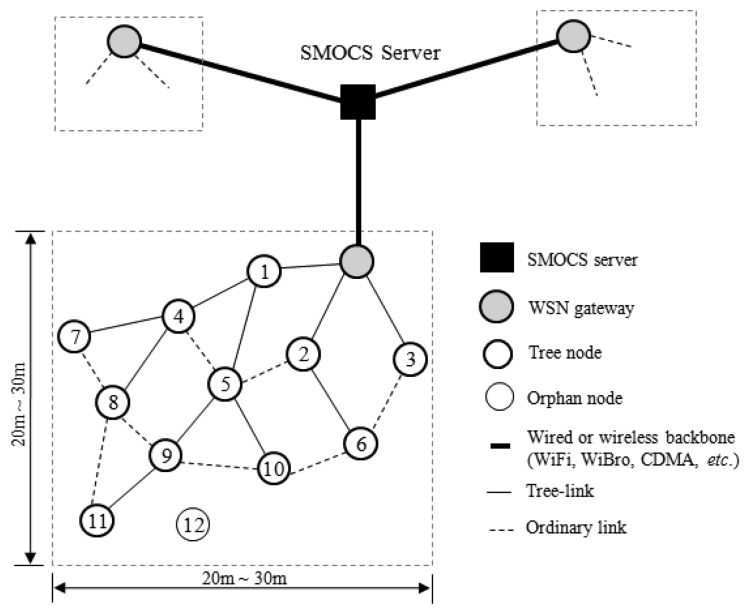
Network model.

Figure 1 shows a typical network model for the SMOCS that consists of a SMOCS server, WSN gateways or sinks corresponding to the number of LPG tanks, and a number of sensor communication devices (sensor nodes). The server is wall-powered, sensor node is battery-powered, and the gateway can be either wall-powered or battery-powered. The server collects data from sensor nodes in the target environment, and analyses these data to judge whether or not a dangerous situation has occurred. Then it can send a command to a sensor node or an actuator in order to warn of the situation. The gateways connect WSNs to a backbone network that can be WiFi, WiBro and/or CDMA. Each sensor node has one wireless communication module, one battery module, and multiple sensor modules which can be switched on or off individually to conserve power. A sensor node senses data from environment periodically or by a request and sends it to the server via a WSN gateway.

A node can be mobile. Sometimes, a link can be broken because of node failure, battery depletion, or the intervention of some communication obstacle. Two nodes which can communicate normally within their mutual transmission ranges are said to have a *link*. This kind of network can be represented as multiple trees, each being originated from a gateway. A node is said to be a *tree-node* if it belongs to a tree. Otherwise, it is an *orphan*. A link between a node and its parent is specially called a *tree-link*. We assume that a node generates at most one data packet to transmit in each round of data collection. Figure 1 shows a network model that consists of three local sensor networking groups. The solid lines and the dotted lines indicate tree-links and ordinary links, respectively.

### 2.2. Notations and Definitions

For more convenience, we use some notations and definitions as follows.

- *h_i_*: Tree depth of node *i*
- *T(i)*: A set of nodes that belongs to a tree whose root is node *i*
- *p(i)*: A parent of node *i*
- *ch(i)*: A set of children of node *i*
- *C_i_* or $C_{i}^{p(i)}$: A control slot demand of node *i*
- *D_i_* or $D_{i}^{p(i)}$: A data slot demand of node *i*

*Definition 1: A control slot* is a time slice that is used exclusively by a node to send one control or command message and a *data slot* is a time slice used exclusively by a node to send one data packet.

*Definition 2*: Given a tree, a *control slot demand* of a node is the sum of the number of control slots requested by its children and the number of control slots it needs to forward one control message from the sink to its children in one cycle. The control slot demand of node *i* with respect to its parent *p*(*i*), denoted by *C_i_* or $C_{i}^{p(i)}$, is given as follows:

(1) $$C_{i}^{p(i)}=C_{i}=\{\begin{array}{cc} \underset{k\in ch(i)}{\text{∑}}C_{k}^{i}+1 & \text{if nodei is not a leaf node}\\ 0 & \text{if nodei is a leaf node} \end{array}$$

Each node *i* calculates *C_i_* recursively, starting from the leaf nodes and moving up to the sink. A leaf node *i* does not need to forward a control message, thus *C_i_* = 0.

*Definition 3*: A *data slot demand* of a node is the sum of the number of data slots requested by its children and the number of slots it needs to forward all data packets in its sub-tree to its parent in one cycle. Thus, the data slot demand of node *i* with respect to its parent *p*(*i*), denoted by *D_i_* or $D_{i}^{p(i)}$, is given as follows:

(2) $$D_{i}^{P(i)}=D_{i}=\{\begin{array}{cc} \underset{k\in ch(i)}{\text{∑}}D_{k}^{i}+\left|T(i)\right| & \text{if nodei is not a leaf node}\\ 1 & \text{if nodei is a leaf node}\\ \underset{k\in ch(i)}{\text{∑}}D_{k}^{i} & \text{if nodei is a}\sin\text{k node} \end{array}$$

Each node *i* calculates *D_i_* recursively, starting from the leaf nodes to the sink. A leaf node needs only one slot to forward its own data packet, thus *D_i_* = 1. An intermediate node has to forward |*T*(*i*)| data packets (its own one plus the data packets received from its descendants) to its parent and also has to give out the slot demands of its children.

### 2.3. Motivation

#### 2.3.1. Problem Identification and Design Approach

**Figure 2.**
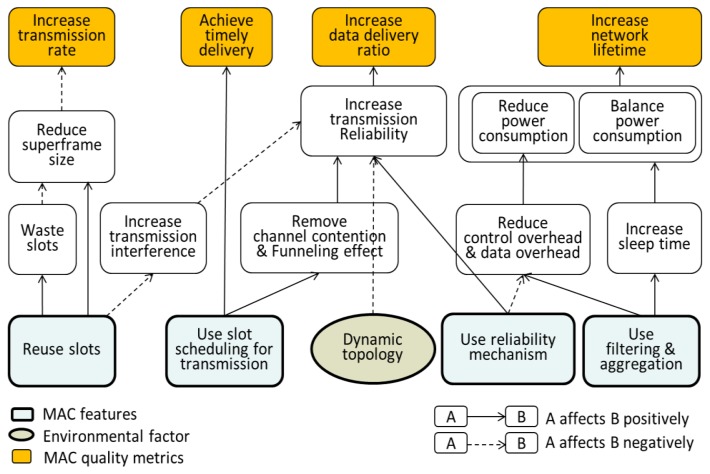
A cause-and-effect analysis graph for MAC features and their effects.

We first examine the MAC features and analyze their effects to set out the design of a new MAC protocol suitable for SMOCS. Figure 2 illustrates a cause-and-effect graph between the possible MAC features and their effects on the performance.

Firstly, the use of the TDMA method eliminates channel contention and funneling effect, thus enabling the timely delivery of packets and increasing transmission reliability. However, it tends to degrade throughput in low traffic networks and needs global time synchronization. In addition, some applications need a bi-directionally reliable communication; however, producing a slot schedule for bidirectional communication is not easy or increases the complexity of any scheduling algorithm.

Secondly, several MACs employ a slot reuse scheme to increase channel utilization and thus to reduce the size of superframe. However, a slot scheduling scheme to reuse slots produces some slots which are not used by any node, thus wasting slots [23]. The significant shortcomings lie in that the slot reuse incurs transmission interference since the interference range is longer than the transmission range of radio signal and increases transmission interference with node mobility, thus invalidating the slot schedule. A partially invalidated schedule is almost impossible to be recovered since the recovery has to take the positions of nodes related to slot reuse into consideration. Thus, a global slot scheduling may have to be performed frequently. Furthermore, the effect of slot reuse in small sized networks is trivial or little effective since it requires an appropriate hop distance between two nodes that use the same identical slot. In TreeMAC [18], the slot reuse becomes effective only when tree depth is greater than and equal to five. Furthermore, its effectiveness is offset by the interference irregularity [24].

Thirdly, the transmission of data within the assigned slot may fail in wireless networks due to link failure caused by node mobility, the distortion of time synchronization, or the environmental factors such as time-varying channel condition, communication obstacles, node failure, and battery depletion. Thus a reliability mechanism using the control messages such as RTS, CTS, and ACK is needed to check whether data was transmitted successfully or not; however it would increase power consumption by the increase of overhead. According to the studies [3,25], this overhead corresponds to 40%∼75% of the channel capacity because data packets are typically very small in sensor networks. A good filtering and aggregation technique can alleviate this problem by reducing a lot of MAC control messages as well as the headers and trailers of data packets.

Lastly, since nodes closer to a sink have to process more data, an ordinary MAC protocol can cause a significant imbalance of power consumption and thus shorten network lifetime. Data filtering and aggregation can be one useful method to alleviate the problem; however, it has to be accompanied by a slot assignment technique that is delicately designed to make filtering and aggregation effective.

Each of the features has a tradeoff; however, considering our objective to design a MAC protocol that ensures reliable transmission and timely delivery in dynamic WSNs to be deployed in the harsh environment, we use TDMA, the reliability mechanism, and a filtering and aggregation technique with elimination of slot reuse. The removal of slot reuse tends to make a MAC protocol robust against node mobility since every slot in a generated schedule is unique over the network; however, it does not help the orphan nodes that have lost their parents to salvage data packets. Thus, for salvaging packets, a MAC protocol either regenerates a slot schedule with modifying topology, or fixes the changed slot demands of nodes after fixing the broken part of topology locally, or can use a spare time utilization scheme that the orphan nodes can utilize the slots which are not used by its neighbors. Note that nodes can produce a lot of spare time either if they are disconnected from some of their children or if a good filtering and aggregation technique is employed.

In our previous study, we proposed the DSA algorithm [23] which does not take into account slot reuse and generates a graceful schedule suitable for filtering and aggregation that takes the pattern of *sending-after-receiving* such that all receiving slots are followed by all sending slots. The increased degree of filtering and aggregation will contribute to not only reducing overall power consumption since it reduces the number of control messages used in the reliability mechanism and the total number of bytes in delivering data packets, but also achieving the balancing of power consumption by granting much higher reduction of power consumption to the nodes closer to a sink. From the above discussion, we summarize the features to be included in a new MAC as well as the features of Funneling MAC [14] and TreeMAC [18] in Table 1.

#### 2.3.2. Effect of Slot Reuse in Small Control Networks

We compare the total number of slots (*superframe*) required for every node to send its data to a sink safely for the FSA algorithm used for TreeMAC and the DSA algorithm used for I-MAC. The superframe size of DSA varies according to tree depth. Assuming that the sink and all sensor nodes are uniformly distributed in a rectangular area of *a* × *a*, the depth distribution of nodes in tree topology can be obtained using the following conditional distribution function of distance *L* between two uniformly distributed nodes which is less than value of *l* [26] as follows:

(3) $$P(L\leq l)=\int_{0}^{l}\int_{0}^{\sqrt{l^{2}-l_{x}^{2}}}\frac{4}{a^{4}}(-l_{x}+a)(-l_{y}+a)dl_{y}dl_{x},0\leq l\leq a$$

(4) $$P(L\leq l)=\int_{0}^{a}\int_{0}^{\sqrt{l^{2}-a^{2}}}\frac{4}{a^{4}}(-l_{x}+a)(-l_{y}+a)dl_{y}dl_{x}+\int_{0}^{\sqrt{l^{2}-l_{x}^{2}}}\int_{\sqrt{l^{2}-a^{2}}}^{a}\frac{4}{a^{4}}(-l_{x}+a)(-l_{y}+a)dl_{y}dl_{x},a\leq l\leq \sqrt{2}$$

where *l_x_* and *l_y_* are the distances between two nodes in the *x-axis* and *y-axis*.

**Figure 3.**
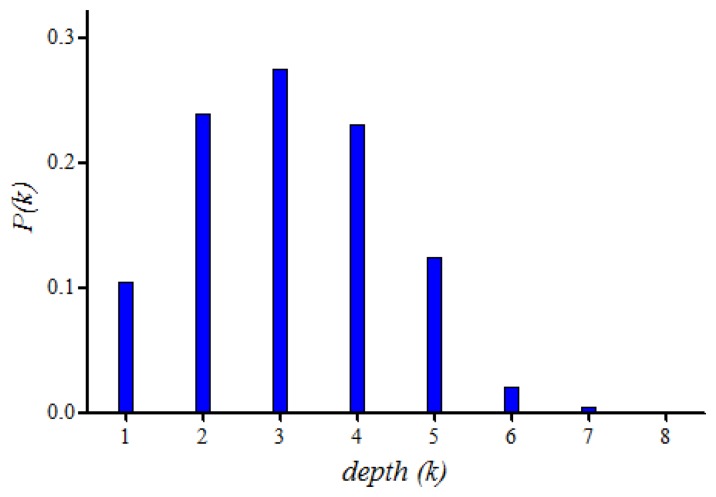
Probability mass function of depth (*k*) (*nNodes* = 25, *a* = 25).

Figure 3 shows the probability mass function *P*(*k*) of random variable *depth (k)* when the number of nodes (*nNodes*) is 25, the transmission range (*r*) of a node is 5 m, and dimension (*a*) is 25 m.

In FSA, each node requires one unique frame of three slots. Therefore, the superframe size of FSA, *s(FSA)*, is given by:

(5) s(FSA)=nNodes×3

On the other hand, according to DSA algorithm, each node *i* (at depth *h_i_*) requires *h_i_* exclusive slots to send one data packet to the sink safely. Thus, the superframe size of DSA, *s(DSA)* is given by:

(6) $$s(\text{DSA})=\sum_{i=I‥\text{nNodes}}h_{i}$$

**Figure 4.**
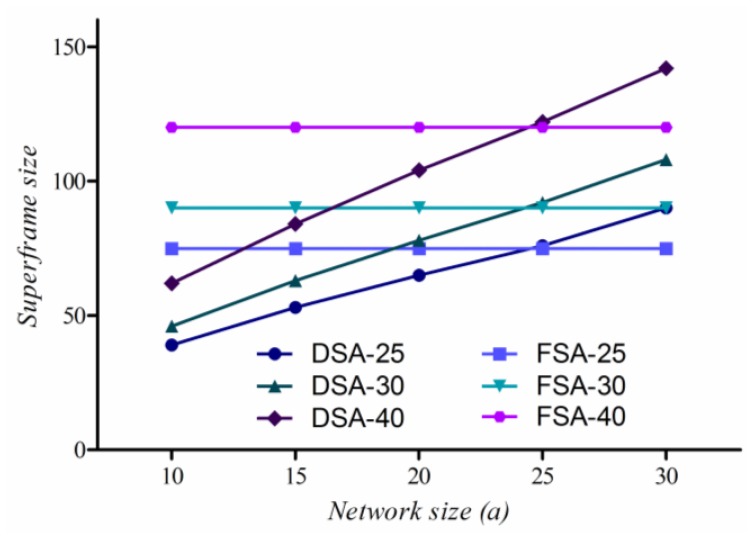
Superframe size according to variation of network size and node density.

By using the probability mass function of Figure 3, we can estimate *s(DSA)*. Figure 4 shows the superframe sizes of DSA and FSA according to variation of nNodes = 25, 30, 40 and *a* = 10 m to 30 m, where the superframe size of DSA increases linearly with the increase of the network size and two graphs cross each other at *a* = 25 m. When a < 25 m, the superframe size of DSA is smaller than that of FSA. However, as *a* becomes bigger, we may have to increase either the number of nodes or the transmission range to prevent network partition, resulting in surely pushing the crossing points to the right. Thus, we conclude that the reduction by slot reuse is trivial when the size of network is small.

## 3. I-MAC Protocol Description

### 3.1. Protocol Structure

**Figure 5.**
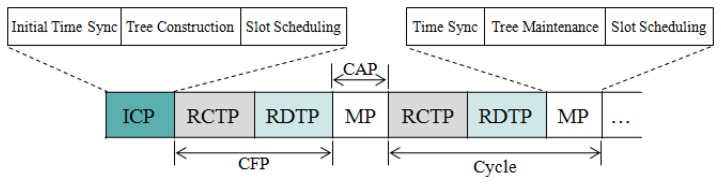
Protocol Structure.

The protocol structure consists of the Initial Construction Period (ICP) and the repeating cycle that includes the Reliable Control Transmission Period (RCTP), the Reliable Data Transmission Period (RDTP), and the Maintenance Period (MP). The former two belong to the Contention-Free Period (CFP), and the latter one belongs to the Contention Access Period (CAP) as illustrated in Figure 5.

In the ICP, initial time synchronization, tree construction, and slot scheduling are performed. The slot scheduling allocates sufficient slots to each node for sending control messages and data packets in every cycle. In the following RCTP and RDTP, the control messages are delivered to either all nodes, a group of nodes or a particular node and data packets are delivered from sensor nodes to a sink, respectively according to the slot scheduling scheme. In the MP, the maintenance activities such as time synchronization, tree maintenance, and slot scheduling are performed.

### 3.2. Time Synchronization

Time synchronization is performed at the beginning of the ICP and MP per the multiple of *k* cycles. Among the existing solutions for time synchronization [27–29], we employ the Flooding Time Synchronization Protocol (FTSP) [28] which achieves high precision time synchronization by utilizing MAC layer time stamping and comprehensive error compensation including linear regression for clock skew estimation.

We use a synchronization message, *SYNC* = (*sinkId*, *sender*, *seqNum*, *globalTime*), where *sinkId* is the ID of the sink node, *sender* indicates the sink or rebroadcasting node, *seqNum* is the sequence number generated by the sink to deal with redundant messages, and *globalTime* is the current time of the sink that is estimated by a sender when *SYNC* is broadcasted or rebroadcasted. The sink broadcasts *SYNC* to initiate time synchronization. Upon receiving *SYNC*, a node obtains the local time that refers to the same instant as global time in *SYNC* from the viewpoint of its local clock. Therefore, *SYNC* provides a synchronization point being a pair <global time, local time> for each receiver. The difference between the global time and the local time of the synchronization point becomes the clock offset of the receiver and the sink. Since the offset is not constant due to clock drift, linear regression method is used to compensate for clock drift. Each node maintains the regression table including eight data points where each data point is a pair <*offset*, *LT*>, *LT* is a local time updated upon receiving a new *SYNC* message. The linear equation for the regression of *offset* on *LT* is as follows [30]:

(7) $$\text{offset}=\bar{\text{offset}}+{\text{skew}}^{*}(LT-\bar{LT})$$

where:

$$\text{skew}=\frac{\sum_{i=1‥n}(LT_{i}-\bar{LT})(\text{offset}-\bar{\text{offset}})}{\sum_{i=1‥n}{\left(LT_{i}-\bar{LT}\right)}^{2}},\bar{LT}=\frac{1}{n}\sum_{i=1‥n}LT_{i},\bar{\text{offset}}=\frac{1}{n}\sum_{i=1‥n}{\text{offset}}_{l}\;\text{and}\;n=8.$$

Therefore, given *LT*, a node can estimate the *global time (GT)* of the sink by:

(8) GT=LT+offset

### 3.3. Tree Construction and Maintenance

#### 3.3.1. Link Quality Estimation

In tree topology, the successful packet transmission is normally affected by the reliability and goodness of the link between two communication nodes. Therefore, in this work, we take link quality into consideration in the process of tree construction. The purpose is to build the reliable tree in which all tree links are bi-directionally reliable to enable the high successful peer-to-peer packet transmission. However, radio links are known to be unreliable, as their behavior unpredictably varies over time and space. The quality of the radio links greatly impact the efficiency of network performance. Therefore, many link quality estimators have been proposed recently and can be classified as either *hardware-based* or *software-based*.

*Hardware-based estimators* exploit information from the radio module directly, by measuring the physical characteristics of received packets, such as Received Signal Strength Indicator (RSSI), Link Quality Indication (LQI). RSSI, with the range of [−100, 0] dBm, is an estimate of signal power which varies according to the distance between a sender and a receiver, transmitting/receiving power, and noise at the receiver; however, it does not care about the quality or correctness of the signal [31], whereas, LQI, with the range of [50, 110], indicates the quality of a received packet, that is given an average correlation value based on the 8 first symbols following the Start Frame Delimiter (SFD) of the IEEE 802.15.4 frame [32]. The major advantage of the hardware-based estimators is that they do not require any calculation since they are performed by the receiver hardware. Moreover, the metrics can be collected from any type of received packets, either unicast or broadcast communication. These metrics depend highly on the accuracy of the hardware. Additionally, the measurements are performed on received packets, the hardware-based estimators cannot exploit any information from packet losses, and become out of operation when there are many packet losses in a radio link. They also do not take into account the asymmetry of links.

*Software-based estimators* require a mechanism to predict the link quality by exploiting information that is independent of the hardware. Typically, based on the reception of packets, link estimators can predict the variation of link quality. Most software-based estimators enable to either count or approximate the packet reception ratio or the average number of packet transmission/retransmission. Packet Reception Rate (PRR) which is the ratio of the number of packets received at the receiver to the number of packets transmitted at the sender can be used to estimate the link quality [33]. However, PRR needs frequent packet exchange in order to update information for the link quality estimator. Moreover, PRR does not reflect the time-varying environmental factors promptly since it is based on some amount of packets transmitted over the corresponding link. Thus, PRR cannot tell a good stable link (a link whose quality remains in good state under external effects) and a good unstable link (a link whose quality is easily affected by the minor environmental change) [31].

**Figure 6.**
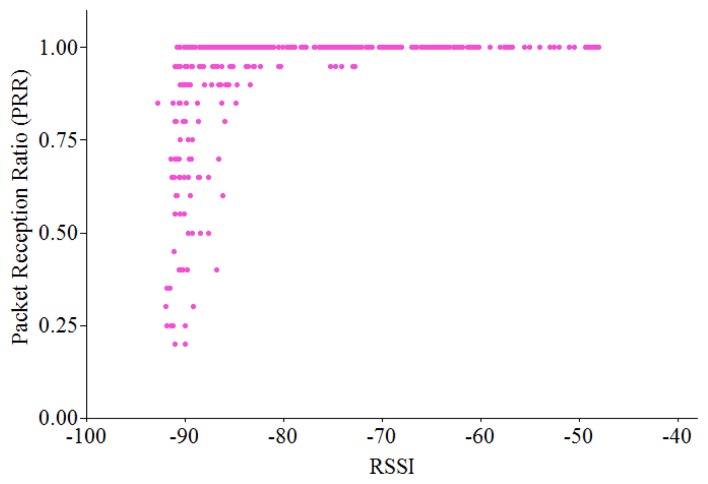
Relationship between RSSI and PRR.

**Figure 7.**
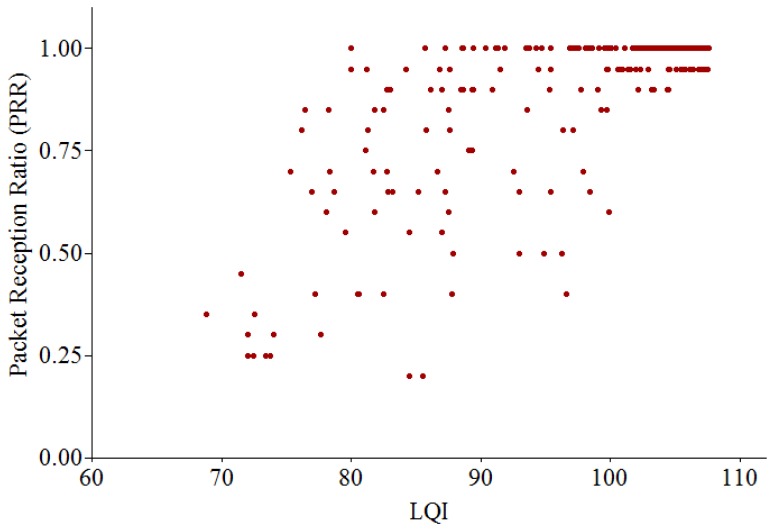
Relationship between LQI and PRR.

We performed several experiments to evaluate the effectiveness of RSSI, LQI and PRR in link quality estimation using TelosB motes running TinyOS 2.1 in an indoor office. One pair of nodes (sender and receiver) is used for data collection. In each experiment, the sender transmits a burst of 1,000 consecutive packets to the receiver with the transmitting power level of −25 dBm. We observe the variation of PRR, RSSI and LQI values for each received packet and calculate their mean values for each experiment. Then we repeated the experiment by changing the distance between the sender and the receiver, increasing 0.1 m each time. As shown in Figure 6, RSSI can only differentiate between the very good links and the rest ones while it is hardly distinguishable between bad, average and good links. Figure 7 shows the relationship between LQI and PRR.

We can see that it is hard to classify between bad, average and good links. It is shown that when LQI is less than 100, a link with PRR = 0.30 can be confused with a link with PRR = 0.90, since they might have the same value of LQI. Consequently, each individual metric (RSSI, LQI or PRR) cannot classify the entire spectrum of link qualities accurately.

To overcome the disadvantage of using individual metrics, the use of three metrics together would increase the accuracy of link quality estimation. The paper [34] proposed a method that combines three link quality metrics (RSSI, LQI and PRR) into a consolidated metric, referred to as *linkq* in this paper:

(9) $$\text{linkq}=\sqrt{{\bar{\text{RSSI}}}_{w}^{2}+{\bar{\text{LQI}}}_{w}^{2}}$$

where $\bar{{\text{RSSI}}_{w}}=\frac{\sum_{k=1}^{m}\left({\text{RSSI}}_{k}+100\right)}{n}$, *and* $\bar{{\text{LQI}}_{w}}=\frac{\sum_{k=1}^{m}{\text{LQI}}_{k}}{n}$, *m* is the number of received packets, and *n* is the number of transmitted packets (0 < *m* ≤ *n*). Using this *linkq*, we judge a link is *reliable* if it is greater than a specified threshold constant, *RLink_Threshold*.

**Figure 8.**
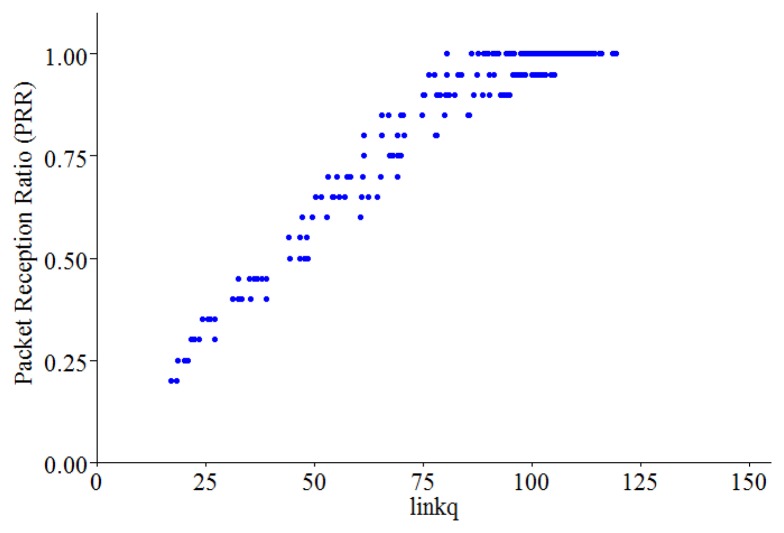
Relationship between linkq and PRR.

Figure 8 shows the relationship between *linkq* and PRR. We use an observation window of 20 packets. From this observation, we can see that *linkq* has a more linear relationship with PRR, since it combines the information embedded in all the individual metrics. This result shows that *linkq* can provide a quick and reliable assessment of the link quality.

#### 3.3.2. Identification of the Bi-Directionally Reliable Link

Most existing research on WSNs assumes that a wireless communication link is a bi-directional and reliable link. It is assumed that a pair of nodes can exchange data each other and the quality of link between them does not change during the operating time. However, in a real scenario, a link may not be bi-directional or its quality may vary due to different transmission powers and antenna gains of nodes, and the effects of path loss, shadowing, fading or external interference. Therefore, for reliable communication, a bi-directionally reliable link needs to be identified.

**Figure 9.**
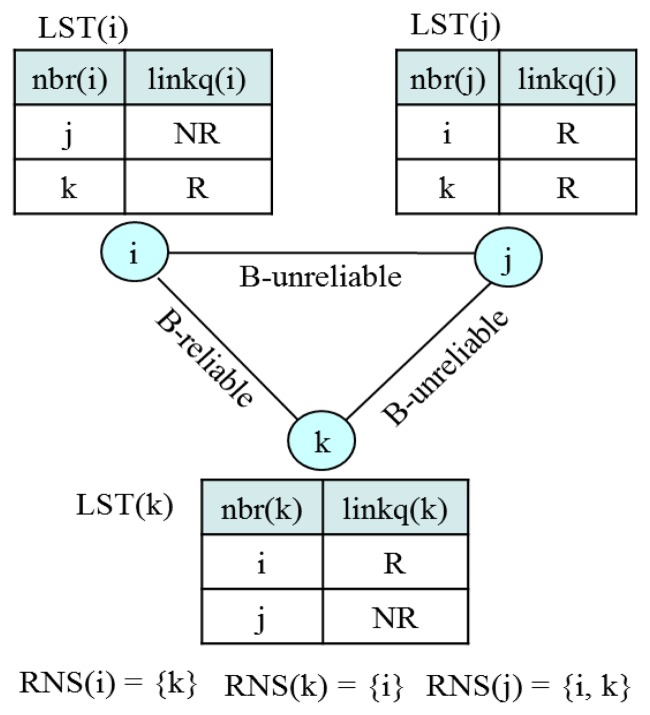
Example of link state table (LST).

We define link (*a*, *b*) to be *bi-directionally reliable* (*B-reliable*) if and only if two directional links from *a* to *b* and from *b* to *a* are *reliable*. Some approaches detect the bi-directional link by explicitly exchanging control messages [35]. However, this causes high overhead and lowers detection reliability since the control messages can be lost due to collision or some other reasons. Hence, in this paper, we propose a new approach to identify the bi-directionally reliable link. Every node *i*, maintains its *link state table*, *LST(i)* = *(nbr(i)*, *linkq(i))*, where *nbr(i)* denotes a neighbor of node *i* and *linkq(i)* indicates whether link (*nbr*(*i*), *i*) is *reliable (R)* or *not reliable (NR)*. Then, let us define a *reliable neighbor set (RNS(i))* as a set of neighbors who is reliable to node *i*. Then, every node *i* is required to include *RNS*(*i*) in a control message. Thus, *LST(i)* is updated whenever node *i* receives or overhears any control message from its neighbor. If a node does not receive or overhear any control message from a reliable neighbor within a specified time bound, *LST_UpdBound*, it removes the neighbor from its neighbor list. Then, a node *i* that has obtained the *RNS(j)* from node *j* determines link (*i*, *j*) to be *B-reliable* if entry *j* of *LST(i) is R* and *i* ∈ *RNS*(*j*). Figure 9 illustrates the determination of a *B-reliable* link.

#### 3.3.3. Tree Construction with Time Synchronization

In the tree construction process, some messages are used as follows:

- TCR = (*sinkId*, *seqNum*, *RNS*, *globalTime*): A sink broadcasts Tree Construction Request (TCR) message to initiate tree construction process, where *sinkID* is an ID of the sink, *seqNum* is a unique message number, *RNS* is a set of neighbors that are reliable to the sender of the message, *globalTime* is used for time synchronization.
- J-REQ = (*sender*, *receiver*, *depth*, *RNS*, *globalTime*): An orphan node (*sender*) sends Join REQuest (J-REQ) message to join a member (*receiver*) and *depth* is a hop distance from the sender to the sink.
- J-RES = (*sender*, *receiver*, *depth*, *RNS*, *globalTime*): A member (*sender*) that receives J-REQ responds with Join RESponse (J-RES) message to the sender of J-REQ (*receiver*).

The tree construction process is initiated by TCR issued by the sink. Upon receiving TCR, an orphan joins the sink by issuing J-REQ if the link between them is B-reliable link. Other orphans that have overheard the J-REQ can join the owner of J-REQ by sending J-REQ to that node. If an orphan has overheard multiple J-REQ messages, it selects a node that has a B-reliable link and the shortest distance to the sink. If there is no B-reliable link, it joins any node in an arbitrary manner. This process repeats until there is no orphan node in the network.

To prevent the collision of J-REQ messages during tree construction, each node sets a timer, *join delay (jdelay)*, as follows before it issues J-REQ:

(10) $$\text{jdelay}=D\times (d_{r}-\max(d_{s},1)+r)$$

where, *d_r_* and *d_s_* are tree depths of an overhearing/receiving node and a sending node of TCR or J-REQ (*d_s_* = *0* if the sender is a *sink*), respectively; *r* is a random number in [0, 1]; and *D* is a small constant delay to be introduced per hop. According to this function, *0* ≤ *jdelay* ≤ *2D*, a node at depth 1 waits for *D* × *r*, and the other nodes wait for *D* × (*d_r_* − *d_s_* + *r*). Thus, this allows the nodes at lower depths to join a tree earlier.

#### 3.3.4. Tree Maintenance

If a node changes its parent, its old and new ancestors have to change their slot demands since they have the number of their descendants changed (*see Definition 3*). This would make the local repair of a tree too complex. In this paper, tree maintenance is not performed until a tree is damaged to some extent. In order to complement this late action, we introduce the *spare time utilization scheme* (which will be presented later) which enables orphan nodes to salvage undelivered packets.

Note that the change of topology in wireless sensor networks occurs quite slowly. Considering this aspect, we allow tree reconstruction to be executed either at the integer multiple of cycles, *treeConsCycle* (= *k* × *cycle*) or at the time when the considerable damage of topology is perceived. To judge the degree of topology damage, a sink counts the number of nodes that has sent data successfully in either an aggregated form or a raw form, given as *nNodesSent*, for each data collection period. We define Data Acquisition Ratio (DAR) as follows:

(11) $$\text{DAR}=\frac{\text{nNodesSent}}{\text{nNodes}}$$

If *DAR* is less than or equal to *DAR_Threshold*, the sink judges that topology has been destroyed enough to reconstruct a tree. Then, it broadcasts TCR during the following MP.

### 3.4. Slot Scheduling

A slot scheduling algorithm consists of a Slot Demand Calculation (SDC) algorithm and a Slot Demand Assignment (SDA) algorithm. In the SDC phase, every node calculates its slot demands for control slots and data slots that it needs from its parent. In the SDA phase, nodes are allocated the unique control and data slots in which they transmit command or control message and data.

#### 3.4.1. Slot Demand Calculation (SDC) Algorithm

**Figure 10.**
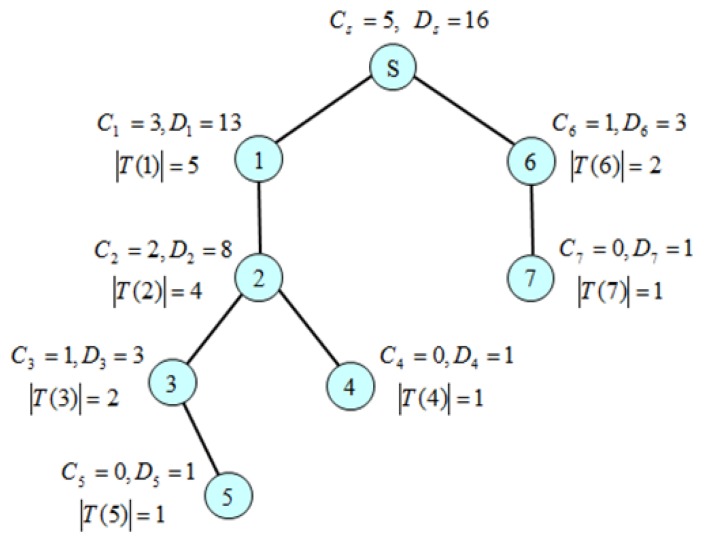
Calculation of control and data slot demands by the SDC algorithm.

The calculation of control slot demand and data slot demand is performed from leaf nodes up to a sink node recursively according to *Definition 2* and *Definition 3*. We use a Slot Demand Calculation message (*SDC_MSG*) expressed as *SDC_MSG* = (*C_i_*, *D_i_*, |*T(i)*|) where *C_i_* and *D_i_* corresponds to the number of control slots and data slots that a node *i* demands from its parent *p*(*i*), respectively. A leaf node sends *SDC_MSG* = (0, 1, 1) to its parent since it does not have to send control message, but has to send one data packet to its parent. An intermediate node *i* waits until it receives all SDC_MSGs from its children, and then calculates *C_i_* and *D_i_*. Then, it sends *SDC_MSG* to its parent. Eventually, the sink *s* can obtain *C_s_* and *D_s_*, assuming that none of *SDC_MSGs* is missing. A timer is used to prevent the locking of the algorithm from the missing of message. Since every node has to send SDA_MSG once, the complexity of message transmission of the *SDC* algorithm is *Θ(n)* when *n* is the number of nodes. Figure 10 shows a slot demand calculation example by the *SDC* algorithm. Since a leaf node does not have to forward a control message, but has to send one data packet, C*_4_* = C*_5_* = *C_7_* = 0 and *D_4_* = *D_5_* = *D_7_* = 1. In this way, *C_1_* = 3, *D_1_* = 13 and *C_6_* = 1, *D_6_* = 3. Finally, we get *C_s_* = 5 and *D_s_* = 16.

#### 3.4.2. Slot Demand Assignment (SDA) and Power Control

Following the completion of the SDC algorithm, the assignment of control and data slots is performed from the sink down to leaf nodes recursively. A node uses a Slot Demand Assignment message (*SDA_MSG*) for slot assignment. For efficiency and reliability of *SDA_MSG* transmission, we use a slotted broadcast mechanism in which a node broadcasts *SDA_MSG* to its children using a control slot which is assigned to itself *on-the-fly* during the execution of slot assignment.

##### (a) Slot Demand Assignment Algorithm

Given that a node has its children list (*i1*, *i2*, …, *ij*, …, *ik*), it produces slot assignments for its children as *SDA_MSG = (schedSlot(i)*, *sysTime())* where *schedSlots*(*i*) = (*c_i1_*, *c_i2_*, …, *c_ik_*), *c_ij_* = *(ij*, *startContSlot(ij)*, *startDataSlot(ij)*) where *startContSlot*(*ij*) and *startDataSlot*(*ij*) indicate the start control slot position and the start data slot position of child *ij*, respectively. Suppose that node *i* has its slot assignment schedule *c_i_* = (*i*, *startContSlot*(*i*), *startDataSlot*(*i*)) which was made by its parent *p*(*i*). Then, *startContSlot*(*ij*) and *startDataSlot*(*ij*) are computed based on *startContSlot*(*i*) and *startDataSlot*(*i*) as follows:

(12) $$\text{startContSlot}(ij)=\text{startContSlot}(i)+1+\sum_{x=1}^{j-1}C_{ix},\;j=1‥k$$

(13) $$\text{startDataSlot}(ij)=\text{startDataSlot}(i)+\sum_{x=1}^{j-1}D_{ix},\;j=1‥k$$

Equation (12) indicates that every node takes the first one control slot among the control slots allocated to it by its parent and then distributes the other ones to its children, implying that it transmits a control message before any of its children (“1” enables this). Equation (13) indicates that a node takes the remaining slots after distributing the data slots allocated to it by its parent to its children. This will create the slot assignment form of *sending-after-receiving* such that all receiving slots are followed by all sending slots to maximize filtering and aggregation.

A node *i* sends *SDA_MSG* using one slot at *startContSlot(i)* scheduled by its parent (a sink uses the first control slot), node *ij*, *j* = *1…k*, will receive it. The detail of *SDA* algorithm with power control mechanism is presented in Algorithm 1. The complexity of message transmission of the *SDA* algorithm is *Θ(n)*, where *n* is the number of nodes except for leaf nodes.

##### (b) Power Control

A node has to know when it wakes up and goes to sleep during control slot and data slot activity. When a protocol enters *RCTP*, every node remains active. If a node *i* finds itself in *SDA_MSG* as one of the destination addresses, it creates *schedSlots*(*i*) for its children and generates *SDA_MSG*. Then, it computes its wakeup time to send a control message *SDA_MSG*, *cWUP*(*i*), and then goes to sleep:

(14) cWUP(i)=startContSlot(i)

Then, it wakes up at the time, *cWUP(i)* and broadcasts *SDA_MSG* using one slot and then enters a sleep state.

For data slots, given that node *i* has its children list (*i1*, *i2*, …, *ik*), it can compute its wakeup time to receive data (*drWUP_j_* (*i*)) from its child *j*, by:

(15) $${\text{drWUP}}_{j}(i)=\text{startDataSlot}(ij)+D_{ij}-\left|T_{ij}\right|,\;j=1‥k$$

Node *i* can also compute the wakeup time for sending data (*dsWUP*(*i*)) to its parent *p*(*i*) as follows:

(16) $$\text{dsWUP}(i)=\text{startDataSlot}(i)+D_{i}-\left|T_{i}\right|$$

**Algorithm 1**. The SDA algorithm.
// *a.b* indicates the element *b* of *a*1: *startContPos* = the start position of C_s_2: *startDataPos* = the start position of D_s_3: **IF** node *i* is sink *s***THEN**4:  **CALL** makeSchedule(*s*, *startContPos, startDataPos*);5: **ELSE IF** node *i* is a leaf node6:  wait for *SDA_MSG (schedSlot(x), sysTime())*;7:  *compute dsWUP(i) and then set timers*;8:  go to sleep until a timer expires;9: **ELSE** // node *i* is an intermediate node10:  wait for *SDA_MSG (schedSlot (x), sysTime())*;11:  *startContPos* ← *schedSlot* (*x*).c*_xi_*. *startContPos*;12:  *startDataPos* ← *schedSlot* (*x*).c*_xi_*. *startDataPos*;13:  *compute cWUP(i) and dsWUP(i), and set timers*;14:  **CALL** makeSchedule(*i*, *startContPos, startDataPos*);15:  go to sleep until a timer expires;16: **END IF**17: **PROCEDURE** makeSchedule(*i*, *startContPos, startDataPos*)18:  *startContPos* ← *startContPos + 1*;19:  **FOR** each node *k* in *ch(i)*20:   add (*k*, *startContPos, startDataPos*) to *schedSlot* (*i*);21:    *startContPos* ← *startContPos* + *C_k_*;22:    *startDataPos* ← *startDataPos* + *D_k_*;23:    *compute drWUP_k_(i) and set timers*;24:  **END FOR**25:  **IF** cWUP(i) expires **THEN**26:    send *SDA_MSG* (*schedSlot (i), sysTime()*);27:  **END IF**28: **END PROCEDURE**


**Figure 11.**
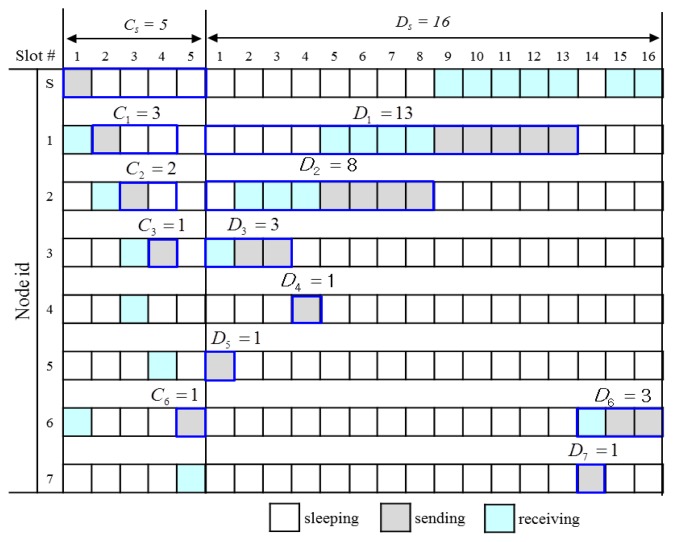
An assignment example of control and data slots by the SDA algorithm.

Figure 11 shows an execution example of the SDA algorithm for the simple tree shown in Figure 10. Since the sink *S* sends only the control message, it takes control slot #1 as the start control slot, but does not need any data slot for transmission. So, it distributes (C_s_−1) control slots and D_s_ data slots to nodes 1 and 6 according to their demands: C_1_ = 3, C_6_ = 1, D_1_ = 13, D_6_ = 3. It sends SDA_MSG = (((1, 2, 1), (6, 5, 14)), sysTime()). Node 1(6) takes slot #2(#5) as the start control slot according to Equation (12) and slot #1(#14) as the start data slot according to Equation (13). Node 1 distributes eight slots to its only one child 2 and reserves |T_1_| = 5 data slots for its transmission from the rear. Furthermore, node 1 wakes up at the slot 5 according to Equation (15) to receive data from node 2 and wakes up at slot 9 (remains waken up in this case) according to Equation (16) to send data to its parent *S*.

### 3.5. Transmission Control

#### 3.5.1. Transmission of Command or Control Message

A control message is not always disseminated to all nodes. If every node rebroadcasts a control message that is destined for one or some nodes, this will cause the unnecessary waste of energy. To control rebroadcasting, each node *i* maintains a set of its descendants, *DS*(*i*) by parsing data packets that its descendants send to the sink periodically. Then, the sink specifies either a unicast address, or a multicast address, or a broadcast address in a control message. If the target address is a broadcast address, every node rebroadcasts the control message, except for the leaf nodes. If the target address is a multicast address, the sink attaches a set of receiving nodes denoted as *G*. Then, a node, say *i*, rebroadcasts the message only if *DS*(*i*) ∩ *G* ! = *ϕ*. If the target address is a unicast address, node *i* rebroadcasts the message only if it finds the target address in *DS*(*i*). Otherwise it discards the message and gets into a sleep state.

#### 3.5.2. Reliable Data Transmission

A node (sender) can start sending data packets (DATA) to its parent (receiver) at the *startDataSlot* computed by Equation (13). However, we cannot guarantee the success of data transmission due to the possible movement of sender and/or receiver, the distortion of time synchronization, time-varying channel conditions, communication obstacles, node failures, and battery depletion. Thus, I-MAC uses three control messages, Ready to Send (RTS), Ready to Receive (RTR) and Acknowledgement (ACK).

**Figure 12.**
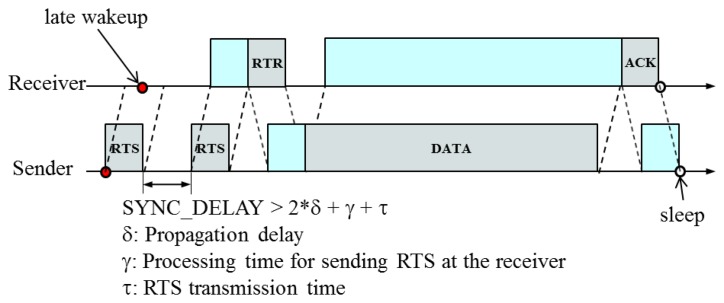
Reliable data transmission scheme.

Considering the drift of the synchronized time such that a receiver wakes up later than a sender, we use the following data transmission procedure: a sender sends RTS to its parent to check if the receiver (its parent) is ready, and then waits either until it receives RTR or for the specified time, *SYNC_DELAY* > 2 * δ + γ + τ, where δ is a propagation delay, γ is a processing delay and τ is a transmission delay. It repeats this process for the specified number of times, *MAX_TIMES* (=2 in this paper) until it receives RTR. If it still does not receive RTR, the sender determines that the corresponding link is broken. If the sender receives RTR, it starts sending DATA to the receiver. During data transmission, DATA also can be lost due to the breakage of the corresponding link. Thus, the receiver responds with ACK to indicate that it has received DATA successfully. Figure 12 illustrates the reliable data transmission scheme.

These MAC control messages may cause quite high overhead if the size of data packet is relatively small. However, we can reduce the number of transmissions and increase the size of packets significantly by achieving the high degree of filtering and aggregation due to the slot demand assignment pattern. This will remove a lot of MAC control messages as well as the headers and trailers of data packets. The most important aspect is that the nodes closer to the gateway can better benefit from the filtering and aggregation. Balancing power consumption in this way will increase network lifetime.

#### 3.5.3. Spare Time (ST) Utilization Scheme

**Figure 13.**
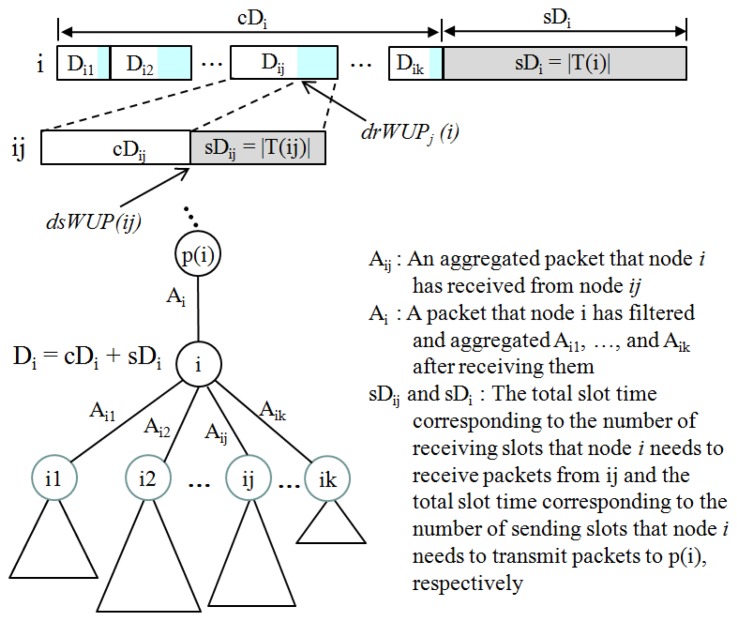
Slot demand structure and relationship between a node and its children.

Suppose that the data slot demand *D_i_* of a node *i* with *k* children, *i1*, *i2*, …, *ik* is represented as *cD_i_* + *sD_i_* where *cD_i_* = ∑*_x_*_= 1..k_*D_ix_* as corresponding to the portion that node *i* distributes to its children and *sD_i_* = |*T_i_*| as the portion that node *i* needs to send packets to its parent according to *Definition 3*. In turn, *D_ij_* = *cD_ij_* + *sD_ij_*, where *sD_ij_* corresponds to the number of receiving slots that node *i* needs to receive packets from its child *ij*. The detailed structure of slot demand between a node and its children is illustrated in Figure 13.

Node *i* is allocated the |T(ij)| receiving slots by the SDA algorithm to receive |T(ij)| packets from its child *ij*. Since node *ij* use filtering and aggregation mechanisms:

(17) $$TL(A_{ij})<sD_{ij}*TL(p)$$

where *p* indicates a single packet and *TL*(*x*) indicates time length of *x*.

If node *ij* does not use *sD_ij_* due to either data aggregation and filtering or the missing of data packets by a link failure to its parent *p*(*ij*) (=*i* in Figure 13), the Spare Time (ST) is made. The ST can be utilized by other neighboring nodes under the permission of the owner.

A node *i* wakes up at *drWUP_j_(i)* defined in Equation (15) at the same time when node *ij* enters *sD_ij_* at *dsWUP*(*ij*) (= *drWUP_j_(i)*) defined in Equation (16), and listens to the channel to receive *A_ij_* from *ij*. Whenever it receives A_ij_, it puts it into a queue. Upon entering *sD_i_*, it performs filtering and aggregation for all packets in the queue and sends a single aggregated packet *A_i_* to its parent *p(i)* using *sD_i_*. In this process, a spare slot can be secured during each sD_ij_, *j* = *1..k*. Assuming that every node sends one aggregated packet to its parent, the *spare slot utilization* scheme is performed by taking two steps—*spare slot acquisition and announcement*, and *spare slot contention*:

(a) Spare time acquisition and announcement
  (*Filtering and Aggregation*) If node *i* has consumed only *x* time units out of *sD_ij_* * *TL*(*p*) to receive *A_ij_* from *ij*, it sends *ACK* = (*ST*) where *ST* = *sD_ij_* * *TL*(*p*) – *x*, in response to the reception of *A_ij_*.
  (*Link Failure*) If node *i* finds the channel idle for a specified time *τ_wait_* after it reaches *drWUP_j_(i)*, it determines that the link to *ij* is broken. It then broadcasts a *spare slot message*, *SS_MSG* = (*nodeId*, *ST*), *ST* = *sD_ij_* * *TL*(*p*) − *τ_wait_*.
(b) Spare time contention
  An orphan that has packet(s) to send remains active until either it is allocated slots by the *SDA* algorithm or it sends packets using *ST*. Upon overhearing *ACK* or receiving *SS_MSG*, if the orphan finds that *ST* is large enough to transmit its packet, it senses the channel. If the channel remains idle for a random backoff time, it sends RTS to the owner of *ST*. If it receives RTR, it transmits the packet and then goes to sleep.

## 4. Implementation and Experiments

### 4.1. Testbed

#### 4.1.1. Implementation

To evaluate the performance of the I-MAC protocol, we conducted experiments with an indoor testbed of TelosB motes running TinyOS 2.1. The TelosB mote uses the Chipcon CC2420 radio [25] which is compliant with the IEEE 802.15.4 PHY layer standard in the 2.4 GHz ISM band with an effective data rate of 250 kbps. The mote uses an 8 MHz TI MSP430 microcontroller with 10 kB RAM. The current draw of TelosB, excluding the radio, is 1.8 mA in active mode and 5.1 μA in sleep mode. The CC2420 radio consumes 23 mA in receiving/listening mode, 17.4 mA when transmitting at 0 dBm, 21 μA in idle mode, and 1 μA in sleep mode.

Since the power consumption of the radio when in receiving/transmitting mode is much greater than that when in sleep mode, the time when a node operates in receiving/transmitting or listening mode should be kept as less as possible. In CC2420, the transmission power can be programmed at 8 discrete levels between −25 dBm and 0 dBm by setting the *TXCTRL.PA_LEVEL* register values from 3 to 31 in steps of 4. In the experiments, the transmission power level is set to level 3 (−25 dBm) and the corresponding radio transmission range is 5 m, approximately. The transmission channel can be selected from 16 channels available (from channel 11 to channel 26) in the 802.15.4 spectrum. However, since some channels of 802.15.4 may overlap with those of 802.11, we use channel 26 to eliminate interference in the presence of the 802.11 WIFI signal. The key parameters are listed in Table 2.

If two neighbor nodes generate two different data packets with the similar oxygen levels whose difference falls within the threshold, a node that receives two data packets can filter one out. Every node generates data packet that includes an integer *key* generated by the following random function:

(18) $$\text{key}=\text{rand}\left(1,\max(1,k\times \frac{\text{nNodes}}{\left|ch(s)\right|})\right),\;k=[0,1]$$

The *k* value affects the duplication intensity of data packets: if *k* gets smaller, the number of packets with the same *key* will increase, thus increasing filtering opportunity. We divide the upper range by |ch(s)| to increase the reasonableness of this function, considering that two or more packets with the same *key* will not be filtered out if they are spread across the different sub-trees of the sink *s*.

#### 4.1.2. Test Scenarios

**Figure 14.**
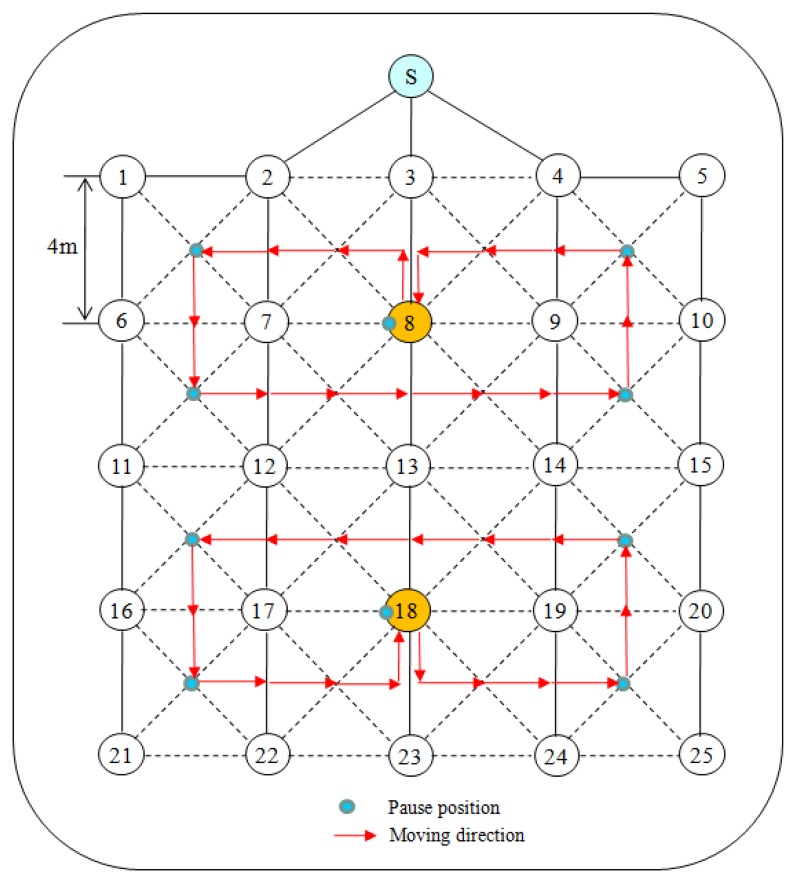
The dynamic deployment scenario.

The following two scenarios were used to evaluate performance of the protocols:

- Scenario I: Static deployment scenario
  Twenty five sensor nodes are randomly distributed in an indoor office with the dimension of 20 m × 30 m. The sink is placed at the top center of the experimental area and all nodes are immobile. However, during the experiment time, the network topology can be changed due to the effects of time-varying channel conditions, such as path loss, shadowing, fading or external interference.
- Scenario II: Dynamic deployment scenario
  Twenty five sensor nodes are artificially distributed in a 5 × 5 grid of the experimental area, making equal distance of about 4 m between the neighbor nodes in vertical or horizontal directions as shown in Figure 14. The solid lines indicate tree links and the dotted lines indicate ordinary links between two neighbor nodes.
  For the distribution of nodes shown in Figure 14, a tree topology can be formed in many different ways since the transmission range and quality of a mote differs according to the directions and time. The given tree topology is just one of them. Two nodes, 8 and 18, travel along the arrowed trajectories. Each of them stays at the center of the corresponding trajectory initially. It then moves to the point indicated by a small shaded circle at the speed of 1 m/s and then stays there for 1 min before it resumes the movement. The nodes repeat this movement process until the end of experiment.

### 4.2. Experiments with Scenario I

#### 4.2.1. Packet Delivery Ratio

Packet Delivery Ratio (PDR), given as the ratio of the number of data packets received at the sink to the number of data packets transmitted by all nodes in the network, is one of the most important performance metrics in evaluating a reliable MAC protocol.

**Figure 15.**
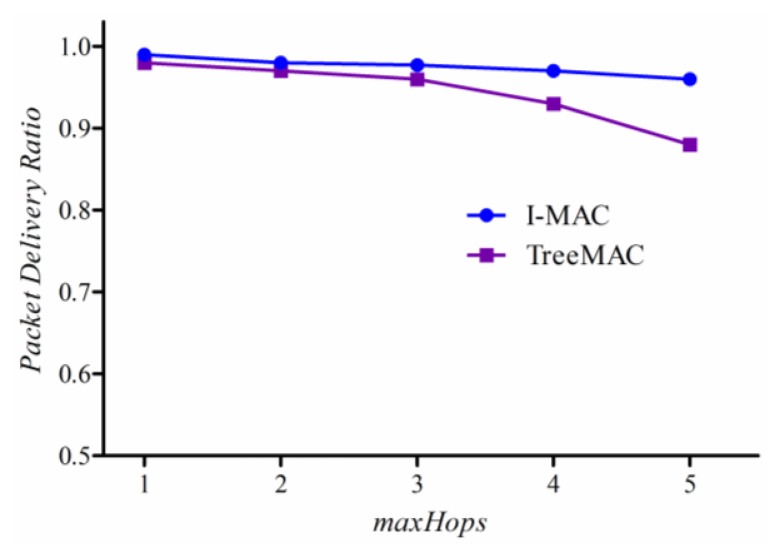
Packet delivery ratio.

Figure 15 compares PDR of I-MAC and TreeMAC according to variation of *maxHops* (the maximum distance in hops from sources to a sink). When maxHops ≤ 3, I-MAC and TreeMAC achieve high PDR with a little difference because no nodes transmit data packets at the same time that can cause interference (slot reuse does not apply in TreeMAC when maxHops ≤ 3). The packet delivery ratio of I-MAC sustains over 0.96 overall while that of TreeMAC decreases down to 0.87 as maxHops increases up to 5.

This improvement comes from two aspects. Firstly, for the reliable and effective delivery of a slot scheduling message, we employ the slotted broadcasting method from our previous paper [30] to deliver *SDA_MSG* in a contention-free manner. Secondly, I-MAC enables an efficient aggregation and filtering method that contributes to reducing the number of data packet transmissions in I-MAC. On the contrary, TreeMAC that uses a slot reuse technique assumes that the vertical three-hop neighbors do not undergo interference; however, the interference is inevitable since the interference range is always farther than the communication range.

These results show that it is almost impossible to achieve 100% of packet delivery ratio in real applications in WSNs, even in one-hop network (as also shown in Figures 6, 7 and 8). A sender may not transmit data packets successfully to a distant receiver due to the effects of signal attenuation, path loss, shadowing, fading or external interference, *etc*. To mitigate this problem, I-MAC employed a mechanism to improve the reliability of packet transmission by building a tree topology with the bi-directionally reliable links.

#### 4.2.2. Packet Loss Rate

We measure a *packet loss rate per node* which is given as the ratio of the number of lost data packets to the number of packets transmitted at each node. For both protocols, we used ACK to confirm that the receiver has received data packet successfully.

In this experiment, we generated five different scenarios with increasing *maxHops* for each experiment. First, we generate the scenario with maxHops = 1 where all nodes are located within one hop from a sink. After performing experiment, we changed the topology artificially to have maxHops = 2 and then experimented the protocols again. Continuously, we performed experiment with the scenario of maxHops = 5.

**Figure 16.**
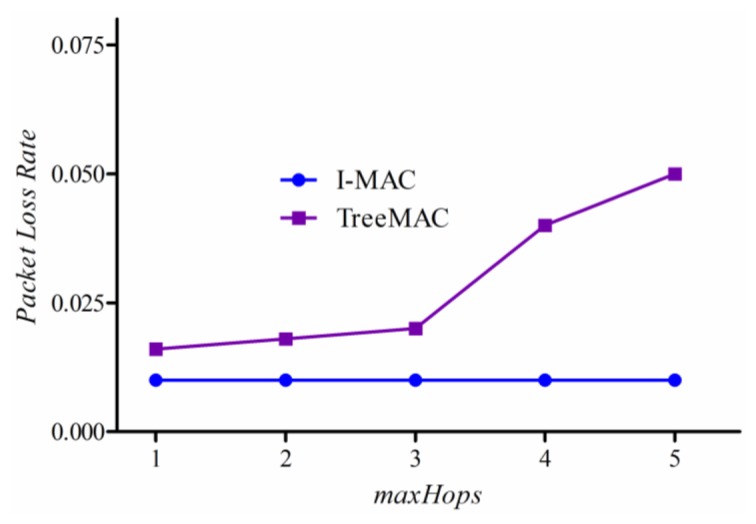
Packet loss rate.

Experimental results are shown in Figure 16. The packet loss rate of TreeMAC increases quickly with the scenario with maxhops = 4 and the scenario with maxHops = 5 since the nodes at depths 1 and 4, and also the nodes at depths 2 and 5 can interfere due to the reuse of slots. Note that the other factors such as node mobility, node failure, and battery depletion were not taken into account in this experiment.

#### 4.2.3. Filtering and Aggregation Capability Index

Transmitting all packets that contain similar information is waste of time and resource. For instance, if the difference of temperatures in any two packets is less than a specified minimum value, *MinDiff*, one packet can be filtered out. Thus, we define *Filtering and Aggregation Capability Index (FACI)* as follows:

(19) $$\text{FACI}=\frac{\text{nBytes}-\text{nBytesRcvdAtSink}}{\text{nBytes}}$$

where *nBytes* indicates the number of bytes of data packets generated in a network and delivered successfully to the sink, and *nBytesRcvdAtSink* is the number of bytes of data packets received at the sink.

**Figure 17.**
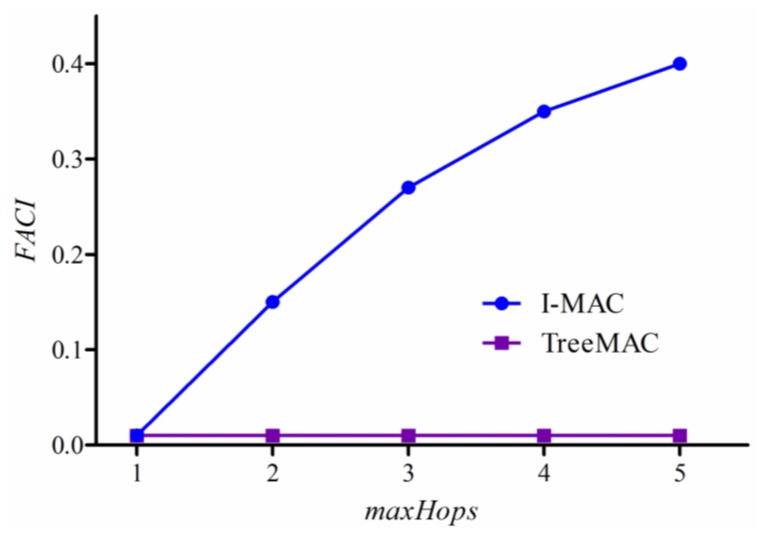
Filtering and Aggregation Capability Index.

Figure 17 shows that I-MAC has a good capability of filtering and aggregation while TreeMAC does not have a notable capability. I-MAC shows the better FACI with the increase of tree depth or *maxHops*, thereby reducing the number of received bytes by 40% with maxHops = 5.

#### 4.2.4. Energy Consumption

According to the CC2420 datasheet [36], the CC2420 radio consumes 23 mA in a receiving or listening mode, 8.5 mA when transmitting at −25 dBm, 21 μA in an idle mode, and 1 μA in a sleep mode. Therefore, to measure the energy consumption of a sensor mote, we count the amount of time that each node has spent in a particular operation mode: sleep, idle, receiving or transmitting. Then, energy consumption of a node is calculated by multiplying the cumulative time stayed at each mode and power consumed to operate the radio in that mode, considering a battery of 3 V. In this way, energy consumption is measured indirectly because of the difficulty in directly measuring the current draw on the physically small and low-power mote.

**Figure 18.**
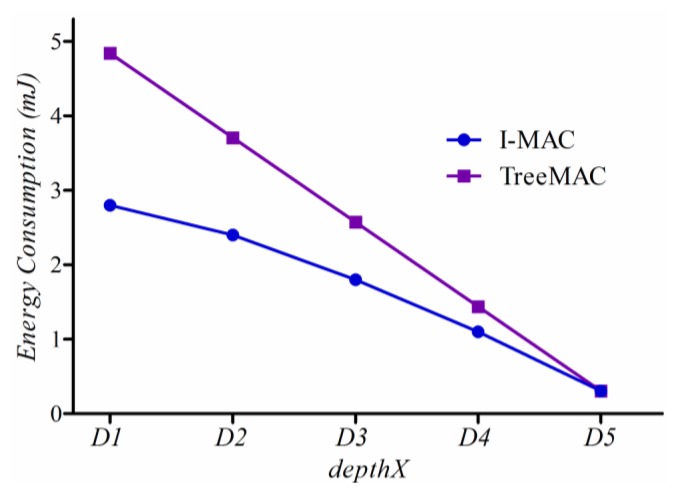
Energy Consumption.

Figure 18 shows how much energy a node located at a particular depth consumes, where *depthX* indicates the distance in hops from the source to the sink. In TreeMAC, energy consumption increases rapidly as the depth of node decreases; whereas, I-MAC has a slow increasing curve due to the superior capability of filtering and aggregation. We can see that nodes located at depth 1 improve energy consumption by more than 40%. We can conjecture that the considerable alleviation of the imbalanced power consumption will increase network lifetime.

### 4.3. Experiments with Scenario II

**Figure 19.**
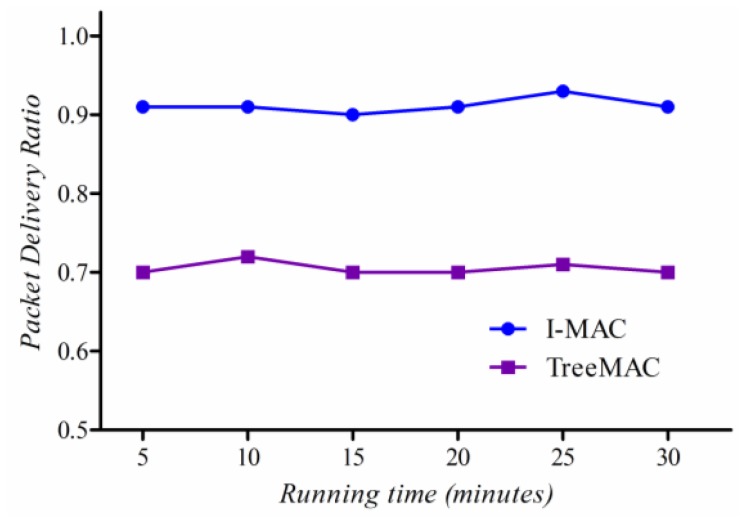
The packet delivery ratio in dynamic topology.

**Figure 20.**
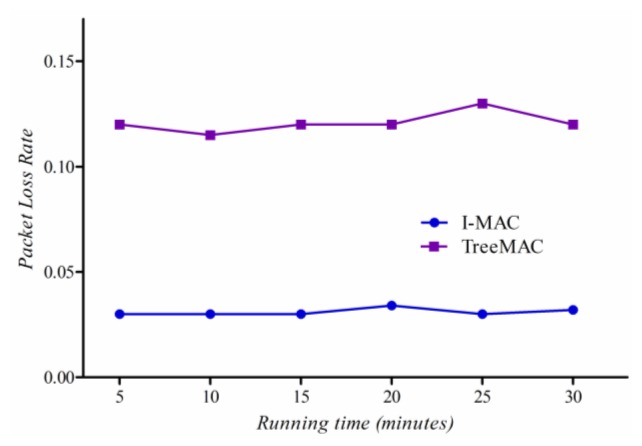
The packet loss rate in dynamic topology.

We compare the reliability of two protocols in Scenario II with some mobile sensor nodes as illustrated in Figure 14. We measured the packet loss and the packet delivery ratio of mobile nodes for each of several 5-minute time windows. Since I-MAC uses the spare slot utilization scheme, and the aggregation and filtering technique, and reliable transmission mechanism, it shows a highly stable packet delivery ratio and a low packet loss rate as shown in Figures 19 and 20. Node that the sender in TreeMAC cannot be aware of a broken link since it does not use the MAC control messages within a slot.

## 5. Concluding Remarks

We have implemented and evaluated a new I-MAC protocol which makes use of the demand-based slot assignment (DSA) algorithm that does not reuse a slot. I-MAC targets a small-sized monitoring and control sensor network of less than 50 nodes that changes in its topology. Since it does not reuse slots, it is free from interference in packet transmission. I-MAC employs a reliable data transmission mechanism within a slot to cope with link failure by node mobility, the distortion of time synchronization, or the environmental factors such as time-varying channel condition, communication obstacle, node failure, and battery depletion. It also uses a spare slot utilization scheme to salvage packets in case of link failure, instead of invoking a costly slot rescheduling. This scheme worked well with the DSA algorithm that enables good filtering and aggregation and thus produces good spare slots. It also includes a method to transmit a command or control messages to some node(s) or all nodes. The experimental results showed that I-MAC could outperform TreeMAC in terms of data delivery ratio, packet loss rate, and energy consumption and balancing in both static and dynamic topologies.

## Figures and Tables

**Table 1.** MAC features for a new MAC.

**MAC Features**	**TreeMAC**	**Funneling MAC**	**New MAC**
TDMA	Yes	Yes	**Yes**
Slot reuse technique	Yes	Yes	**No**
Reliability mechanism	No	No	**Yes**
Filtering and Aggregation technique	Almost impossible	Limited	**High**
Responsiveness to dynamic topology	Low	Low	**High**
Bi-directional capability of communication	No	No	**Yes**

**Table 2.** I-MAC experiment parameters.

**Parameter**	**Value**
Default transmission power	−25 dBm (power level 3)
Channel frequency	2.480 MHz (channel 26)
Radio bandwidth	250 kbps
Sensor model	TelosB
Slot size	20 ms
Frame size (f) (TreeMAC)	3 slots
Cycle size (TreeMAC)	f × n (n = number of nodes)
Data packet length	100 bytes
Dimension	20 × 30 (m^2^)
Number of nodes	1 sink; 25 sensor nodes
Experiment time	3,600 s
MAX_TIMES	2
SYNC_DELAY	1 ms
